# Link between b.c.c.–f.c.c. orientation relationship and austenite morphology in CF8M stainless steel

**DOI:** 10.1107/S1600576724008392

**Published:** 2024-10-01

**Authors:** Maxime Mollens, Adrien Guery, Dominique Loisnard, François Hild, Stéphane Roux

**Affiliations:** a EDF R&D, Site des Renardières, 77818Moret-sur-Loing, France; b Université Paris-Saclay, CentraleSupélec, ENS Paris-Saclay, CNRS LMPS – Laboratoire de Mécanique Paris-Saclay, 91190Gif-sur-Yvette, France; Ecole National Supérieure des Mines, Saint-Etienne, France

**Keywords:** electron backscatter diffraction, EBSD, orientation relationships, dual-phase materials, image processing, segmentation

## Abstract

Slow-cooled CF8M duplex stainless steel is used for critical parts of the primary coolant pipes of nuclear reactors. This steel can endure severe service conditions, but it tends to become more brittle upon very long-term aging (tens of years). Therefore, it is essential to understand its specific microstructure and temporal evolution. As revealed by electron backscatter diffraction (EBSD) analyses, the microstructure consists of millimetre-scale ferritic grains within which austenite lath packets have grown with preferred crystallographic orientations concerning the parent ferritic phase far from the ferrite grain boundaries. In these lath packets where the austenite phase is nucleated, the lath morphology and crystal orientation accommodate the two ferrite orientations. Globally, the Pitsch orientation relationship appears to display the best agreement with the experimental data compared with other classical relationships. The austenite lath packets are parallel plate-shaped laths, characterized by their normal **n**. A novel methodology is introduced to elucidate the expected relationship between **n** and the crystallographic orientation given the coarse interfaces, even though **n** is only partly known from the observation surface, in contrast to the 3D crystal orientations measured by EBSD. The distribution of retrieved normals **n** is shown to be concentrated over a set of discrete orientations. Assuming that the ferrite and austenite obey the Pitsch orientation relationship, the determined lath normals are close to an invariant direction of the parent phase given by the same orientation relationship.

## Abstract 2

The microstructural properties of duplex stainless steel are studied on a wide range of scales using a large electron backscatter diffraction data set. Image analysis techniques are coupled with crystallographic data analysis to extract relevant features from a complex microstructure.

## 1. Introduction

It is well established that the macroscopic mechanical behavior of materials is conditioned by their microstructural properties. For instance, the size, shape and texture of crystals in steels and alloys are first-order parameters in the prediction of their mechanical response (Kim & Thomas, 1981 ▸; Hansen, 2004 ▸; Bouquerel *et al.*, 2006 ▸; Verma & Taiwade, 2017 ▸). In forming processes involving displacive (*i.e.* diffusionless) transformations, the final texture results from the parent phase texture and the symmetry involved in the displacive mechanisms giving rise to the child phase (Bunge *et al.*, 1984 ▸; Bate & Hutchinson, 2000 ▸). Similar conclusions are drawn regarding crystal shapes. The child phase morphology is inherited from the parent crystal orientations and the induced crystallographic consistency between the two phases. All these observations also hold for alloys with diffusion-controlled transformations, although they have received less attention (Maki *et al.*, 1986 ▸; Ameyama *et al.*, 1992 ▸; Monlevade & Falleiros, 2006 ▸).

Several components of the primary coolant system in nuclear power plants are made of alloys involving complex phase transformations, and they are of fundamental importance to ensure safe operation. In their in-service environment, they undergo severe thermal and mechanical loadings for an extended period of time (Bethmont *et al.*, 1996 ▸; Jayet-Gendrot *et al.*, 2000 ▸). The temperature they are subjected to leads to slow microstructural transformations resulting in a modification of their mechanical properties. Hence the impact of thermal aging on the risk of failure must be predicted accurately to assess their integrity for long-term operation (Le Delliou & Saillet, 2015 ▸).

Such materials exhibit a very peculiar dual-phased microstructure formed from an α-ferrite (body-centered cubic, b.c.c.) to γ-austenite (face-centered cubic, f.c.c.) transformation that is responsible for its specific corrosion resistance and resilience properties. This microstructure presents remarkable properties over a broad range of scales, from millimetre-sized polygonal parent grains to the atomic scale where thermal aging occurs. When building a consistent multiscale model, careful investigations of the microstructure are to be carried out. Understanding of its dual-phase layout and crystallographic relationships is required to control the deformation mechanisms occurring during the component lifetime. At first sight, both could be deduced from the crystallographic orientation relationship (OR) between the two phases, on the basis of prior knowledge of the parent phase.

Numerous and rigorous experimental and numerical studies have proven the link between ORs, crystallographic properties and morphology in martensitic steels in which displacive transformation (from γ-austenite to α-ferrite) occurs. Morito *et al.* (2006 ▸) have listed a series of historical reports on this issue, and more recent studies continue to tackle the complex mechanisms of martensite transformation with interpretation of experimental observations (Baur *et al.*, 2019 ▸; Ramachandran *et al.*, 2020 ▸) and numerical simulations (Engin & Urbassek, 2008 ▸; Tateyama *et al.*, 2008 ▸; Malik *et al.*, 2012 ▸; Zhang *et al.*, 2021 ▸). Materials with diffusion-controlled transformations were also studied but to a much lesser extent (Ameyama *et al.*, 1992 ▸; Weatherly & Zhang, 1994 ▸). The literature discussing such transformations often considers synthetic materials having microstructures differing significantly from those of industrial duplex stainless steels used in primary cooling circuits.

The strain resulting from γ-austenite to α-ferrite transformation cannot be accomodated by diffusion only and plastic strain thus occurs. Additionally, the slow cooling carried out to reach the desired microstructural properties favors a gradual transformation with elemental partitioning and ultimately local lattice parameter changes (Self *et al.*, 1981 ▸). These factors forbid recourse to historical microscopy tools and models to perform an accurate analysis of transformation strain and product morphology. Hence, it is necessary to develop an experimental method that provides crystallographic measurements and their statistics to describe the microstructure at the scale of industrial components. In this respect, very high accuracy is not critical because of the intrinsic variability of the local microstructure, but a statistical representativeness is sought. The links between crystal orientation and morphology, as well as the description of the spatial distribution of the two phases, are the first steps in a strategy aimed at proposing a microstructure-based constitutive model.

Tools commonly used for accurate determination of crystallographic parameters may not be suitable for the slowly cooled duplex microstructure. In particular, transmission electron microscopy (TEM) is not appropriate as its characterization cannot safely be scaled up to reach the characteristic scale of the grains. Consequently, mostly electron backscattered diffraction (EBSD) analyses will be used in this paper as they allow for generating the statistics needed to move on to higher scales.

The outline of this paper is as follows. First, material characteristics and experimental characterization methods are described in Section 2. Observations regarding the specific microstructural properties are extracted from EBSD measurements in Section 3. To address the complexity associated with the material’s history, a specific post-processing of EBSD maps is introduced for determining preferential directions of austenite (Section 4). The results are then compared with the ultimate accuracy reachable with such approaches. Last, the previously reported observations are discussed in Section 5 together with perspectives towards future studies.

## 2. Experimental procedures

The studied material is a cast duplex CF8M stainless steel. The samples were taken from an 80 kg cast ingot (∼6.4 dm^3^) using electrical discharge machining far from external surfaces to avoid inhomogeneous zones. The ingot was cast, then air-cooled for approximately 15 days before being heat-treated to 1120°C for 6 h and 20 min, and finally water-quenched. The composition is given in Table 1 ▸ for the major chemical components. For scanning electron microscopy observations and EBSD acquisitions, the samples were mechanically polished up to 0.1 µm grain size and then processed with an oxide polishing solution. Acquisitions were carried out on a Mira3 scanning electron microscope from TESCAN using an accelerating voltage of 30 kV. Diffraction patterns (120 × 120 pixels) were imaged on a phosphor screen and captured on a CCD camera provided by Nordif. The indexing was performed with *OIM* data collection software from EDAX using the standard Hough transform method. All EBSD maps had a 3 µm step size. The open source MATLAB *MTEX* toolbox (Bachmann *et al.*, 2010 ▸) was used for its very convenient and flexible plotting features. Since only cubic structures (corresponding to the 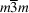 Laue group) are discussed in the following, all classical EBSD maps are colored using a standard inverse pole figure (IPF) color coding using sample normals **z** as a reference. The map coordinates always coincide with the crystallographic orientation reference system. These maps are abbreviated to ‘IPF-Z’ for convenience.

In terms of the volume of data required to extract a statistical description of the microstructure, seven samples taken from different positions in the ingot core were characterized. A 2 × 10 mm map was acquired for each sample. A total surface area of 140 mm^2^ was mapped, which is an order of magnitude larger than the largest microstructure features described in the following.

## 3. Observations

**Figure 1:**
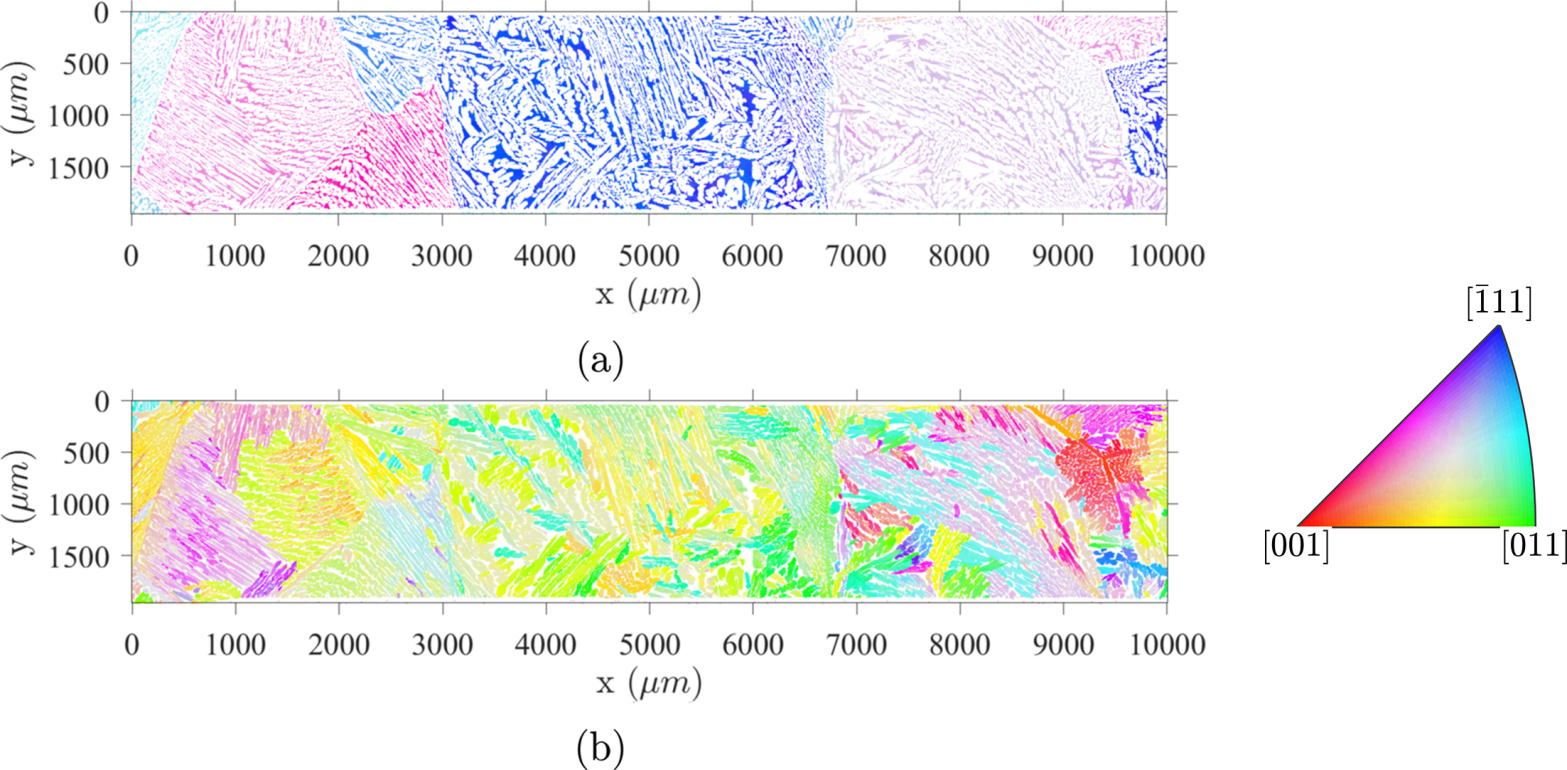
The microstructure of CF8M duplex stainless steel represented by an IPF-Z map. (*a*) An α-phase map displaying large ferritic grains resulting from the first phase transformation. (*b*) The austenitic lath network growing within ferritic grains.

The material obtained from the casting process has two phases, resulting from the partial ferrite to austenite transformation and the solutionizing heat treatment which recovers some of the transformed ferrite during the first cooling step. The analyzed specimens have roughly a 25:75 vol.% ratio for ferrite and austenite, respectively. The resulting microstructure is shown in Fig. 1 ▸. The orientation map of the α-phase reveals the geometry of the primary grains with distinct areas of uniform orientation. Insofar as the ingot is thick, the cooling slow and the samples taken far from the edges, these areas can be considered as resulting from equiaxial grains of random crystallographic orientations. Thus, there is no specific sample symmetry inherited from the casting process Conversely, the γ-phase exhibits elongated lath colonies within which the crystallographic orientations are homogeneous or smoothly varying.

### 3.1. Primary ferrite grain inner structure analysis

In order to quantify possible links between the parent and child orientations, and their correlation with the lath morphology, it is necessary first to estimate the primary phase orientation from where the laths have grown. The remaining α-phase is then utilized to recover the primary grain geometry using, for instance, a crystallographic orientation-based inpainting process (Mollens *et al.*, 2022 ▸). The recovered orientation map displays millimetre-sized polygonal grains resulting from the first phase transformation (*i.e.* liquid to solid). Hence, at each pixel coordinate **x** of the map, the austenite orientation **g**_γ_(**x**) can be connected to its parent phase orientation **g**_α_(**x**) [*i.e.* the misorientation 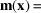

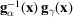
 is measured].

Commonly mentioned orientation relationships (ORs) in b.c.c. ↔ f.c.c. phase transformations in steels include Bain (B) (Bain & Dunkirk, 1924 ▸), Kurdjumov–Sachs (KS) (Kurdjumow & Sachs, 1930 ▸), Nishiyama–Wassermann (NW) (Nishiyama, 1934 ▸; Wassermann, 1935 ▸), Pitsch (P) (Pitsch, 1959 ▸) and Greninger–Trojano (GT) (Greninger & Troiano, 1949 ▸). Experimentally, measured ORs are more than 9° away from B, and most of the time are related to either KS or NW (Verbeken *et al.*, 2009 ▸). These two ORs actually result from experimental observations using X-ray diffraction in the f.c.c. → b.c.c. case. P and GT ORs were identified using TEM approximately 20 years later. Some authors mentioned that ORs in b.c.c. → f.c.c. transformations were far less widely discussed, even though some relatively recent studies on meteorites (Bunge *et al.*, 2003 ▸; He *et al.*, 2006 ▸; Nolze, 2008 ▸; Yang *et al.*, 2010 ▸) and various metallic alloys (Stanford & Bate, 2005 ▸; Fukino *et al.*, 2011 ▸; Rao *et al.*, 2016 ▸; De Jeer *et al.*, 2017 ▸; Haghdadi *et al.*, 2020 ▸; Cai *et al.*, 2021 ▸) have appeared.

The ORs are defined by the set of parallel planes and directions given in Table 2 ▸. The misorientation is then the rotation that maps the crystal directions 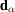 of the child phase to their parallel relatives 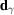 in the parent frame. The misorientation obeying 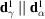 and 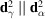 is such that 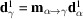 and 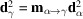. The axis–angle representation of the mis­orientation associated with the parallelism conditions is given in Table 2 ▸. We note that a definition of the orientation relationship based on a misorientation only is somewhat reductive since a non-zero strain is needed to accommodate the crystal atomic spacing.

**Figure 2:**
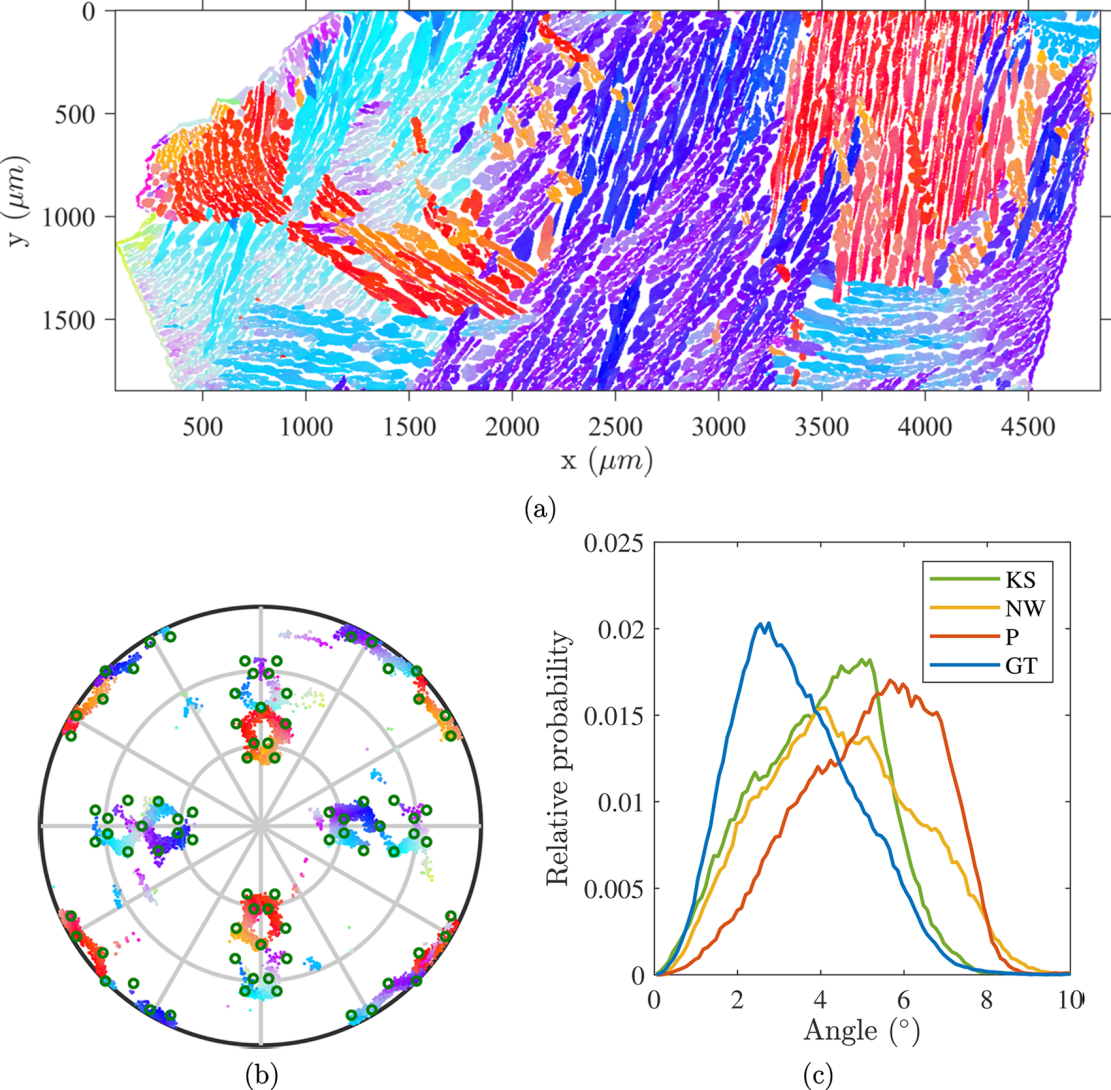
Austenite orientation in a single ferritic grain. (*a*) An IPF-Z map. (*b*) A [111] pole figure in the ferrite crystallographic frame displaying the γ-orientation spread in a single ferritic grain. The orientations are colored using the IPF-Z color coding convention to relate to (*a*). The exact KS variants are plotted in green to emphasize the continuity of the observed OR. Only 1% of measured points of the data present on the spatial map are shown in the pole figure. (*c*) Histograms of rotational angle to the closest variant for four classical ORs in f.c.c. ↔ b.c.c. systems, showing that there is no single representative OR in the CF8M alloy.

Data obtained from a single ferritic grain are shown in Fig. 2 ▸. Fig. 2 ▸(*a*) displays austenite orientations (colored using the standard inverse pole figure code for cubic symmetry) grouped in lath packets with uniform or smoothly varying crystal orientations. These packets are also characterized by a preferred morphology. The comparison with all symmetrically equivalent child orientations (*i.e.* variants) predicted for KS [Fig. 2 ▸(*b*)] and the distance distributions with some reference ORs in Fig. 2 ▸(*c*) highlight the scatter of crystal orientations.

The most widely discussed cases of orientation spread around common ORs involve martensitic steels (Nolze & Geist, 2004 ▸; Cayron *et al.*, 2010 ▸; Yardley & Payton, 2014 ▸; De Jeer *et al.*, 2017 ▸; Hayashi *et al.*, 2020 ▸). Their origin is still under discussion (Bhadeshia, 2011 ▸; Cayron *et al.*, 2011 ▸; Hayashi *et al.*, 2020 ▸) and displacive mechanisms are not expected in the present case because of the very low cooling rate after casting and the long solutionizing process. Instead, significant freedom is given to diffusion mechanisms during manufacture of the ingot. The rotational distance to distinct ORs is then presumably associated with an accomodation to minimize the interfacial energy during precipitate growth following an edge-to-edge sympathetic nucleation process (Ameyama *et al.*, 1992 ▸).

The microstructure of interest presents visible characteristics that differ from diffusion-controlled microstructures studied in the literature. For instance, α–γ stainless steels studied by Maki *et al.* (1986 ▸), Ameyama *et al.* (1992 ▸) and Haghdadi *et al.* (2018 ▸, 2020 ▸) displayed a high density of both intragranular and boundary nucleated precipitates. A fundamental difference with the CF8M alloy is the resulting spatial distribution of child nuclei. The reference materials had a large amount of retained parent phase (around 50%) and a large quantity of child variants are present in each parent grain. The characterized CF8M microstructure appears much closer to the α–γ microstructure of brass characterized by Stanford & Bate (2005 ▸) where the forming conditions had promoted the growth of large millimetre-sized parent grains inside which a few child variant clusters were present. There is also a strong similarity in the variety of morphologies, from globular to sharper-shaped laths, with this Cu–Zn alloy that is missing in the previously mentioned steels. Additionally, all lath clusters seem to have grown from a small number of nuclei forming at former ferritic grain boundaries. This is to be contrasted with the results of Ameyama *et al.* (1992 ▸) who observed a dense array of child nuclei at parent grain boundaries.

**Figure 3:**
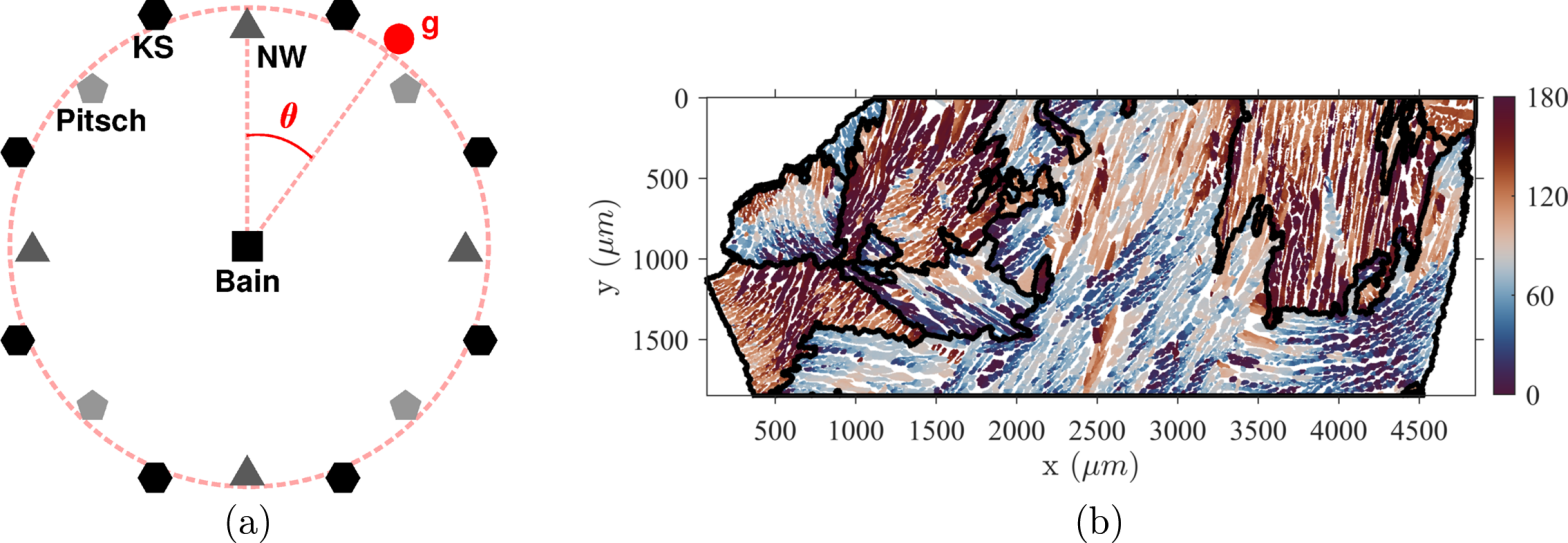
Parameterization of γ-orientations according to their distribution about their corresponding Bain variant in a single primary ferrite grain. (*a*) A schematic representation of θ parameterization for a single Bain variant. The angle is taken between the orthogonal projection **g** and an orthogonal vector taken among three basis vectors of the ferrite grain crystallographic frame. Thus, only the θ variation is indicative of a distribution of orientations around Bain groups. (*b*) A [100] pole figure of γ-orientations in the α-grain reference frame. Orientations are colored according to the value of θ.

Locally measured ORs in a single cluster may vary over a long characteristic length along with morphological changes. This morphological ‘gradient’ is rather linked to a transition between OR variants than to statistical dispersion with respect to a fixed OR. A specific post-processing procedure of orientations was developed to reveal this aspect. Instead of considering the crystallographic distance over the symmetry group, we chose to rely on an image-processing-based interpretation of the distribution of OR variants. Stereographic projections normal to the principal cubic directions of these variants show a nearly circular pattern [Fig. 3 ▸(*a*)] from which the approximate expression ‘Bain circles’ originates. Knowing the Bain circle (*i.e.* Bain variant) on which lies an experimental γ-orientation 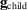, according to its parent α-orientation 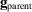, one may parameterize the angular position θ of 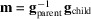 (*i.e.* the associated misorientation) on a circle centered about the Bain variant [Fig. 3 ▸(*a*)]. This step was achieved by projecting the quaternion vector representing **m** onto the plane normal to the Bain variant and computing its polar angle with respect to an arbitrary direction. Hence, knowing 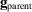 allows one to reduce the description of 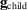 to two parameters, namely the Bain variant and θ. The angle θ computed for austenitic orientations shown in the ferritic grain of Fig. 2 ▸(*a*) is plotted on a [100] spherical projection in Fig. 3 ▸(*b*).

**Figure 4:**
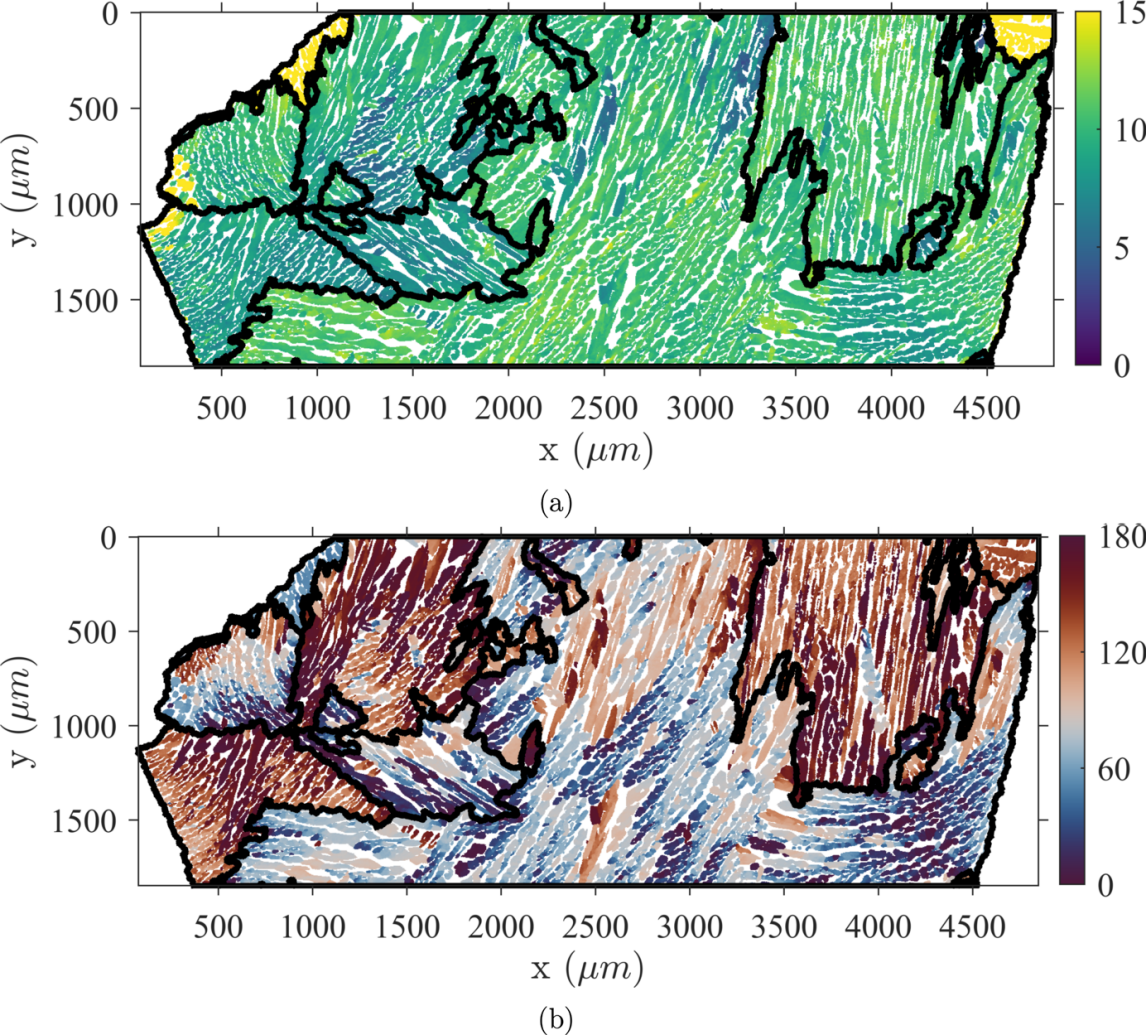
Illustration of the variant crossing phenomenon. (*a*) The crystallographic distance (in degrees) to the Bain OR. Frequently reported KS and NW ORs are at 11.07° and 9.74°, respectively. The map exhibits a much broader scatter. The black boundaries delineate different Bain groups. (*b*) A spatial map colored according to the value of θ. This panel shows that the orientation may cross multiple variants of a given OR in a single Bain group while preserving a spatial continuity at this scale (*i.e.* θ varies much more than 7.54°, which is the smallest Δθ separating two variants in the cited ORs).

Interestingly, the ‘circles’ observed from the present EBSD analysis and shown in Fig. 3 ▸(*b*) display a quasi-continuous distribution about each Bain variant. As shown in Fig. 4 ▸(*a*), many different laths are encountered around each Bain variant, at different (but close) specific distances to the Bain OR. The θ parameterization depicted in Fig. 4 ▸(*b*) reveals an important feature of the γ-orientation distribution. In the ferrite reference frame, the continuous orientation around the Bain circles correlates well with their morphology as observed on spatial maps. In some Bain groups (delimited by black lines on the spatial maps) the θ range is close to 180°, which reflects a transition from a near-horizontal lath shape to a near-vertical lath shape on the 2D section given by the EBSD maps. A 180° θ range also means that multiple ORs are crossed around a Bain group. In other words, a single spatial Bain group may contain multiple variants of a single OR, smoothly connected through progressive orientation changes on the scale of the map. The θ parameterization is only relevant for orientations lying on (or close to) the Bain circles, considering a sensible tolerance regarding the EBSD measurement accuracy. In this example, a small number of regions are far from all Bain variants [distance greater than 15° in Fig. 4 ▸(*a*)]. They result from the phase transformation mechanisms during cooling, and more specifically from phenomena occurring at primary ferritic grain boundaries that are detailed in the following section.

### 3.2. Primary ferrite grain boundary neighborhood analysis

The spatial orientation maps allow one to propose a schematization of austenite nucleation and growth steps. The surfaces describing primary ferritic grain boundaries are first replaced by a ‘thickened’ austenitic surface with the same primitive shape. The nucleation giving rise to these films starts at grain junctions. This observation is supported by the EBSD maps, on which austenite nuclei seem to appear nearly simultaneously at multiple triple points until they meet on the former boundary as a result of the growth kinetics. This observation means that the austenite film around a single ferritic grain is not monocrystalline. Additionally, the child orientation at a junction presumably results from a variant selection mechanism with a *single* grain. Considering that typically four randomly oriented grains meet at a junction in the bulk, a child orientation cannot accurately follow an OR with all of them. The orientation maps reveal that the child nuclei orientations are linked to a single parent grain only and that these orientations are kept by laths growing inside the grains, even though no OR is followed with most of them.

**Figure 5:**
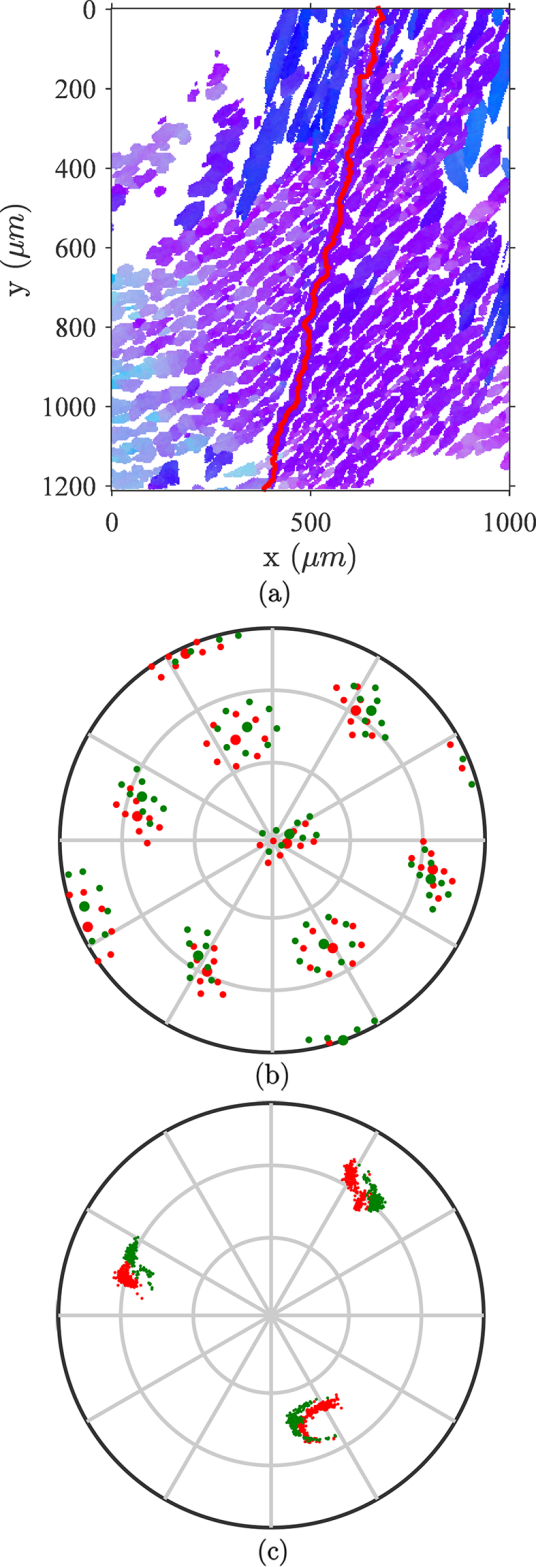
Orientation and morphology for an almost-coherent grain boundary. (*a*) An IPF-Z map. The primary α-grain boundary is plotted in red. The morphology and orientation of austenite appear similar on either side of the boundary. (*b*) A [100] pole figure of the simulated KS and B variants given the primary grain orientations. The red points correspond to the left-hand grain and green to the right-hand grain. (*c*) A [100] pole figure showing actual γ-orientations measured in both grains using the previous color coding.

Since lath packets grow from former ferritic grain boundaries, the latter are classified into two categories, *almost-coherent* boundaries (*i.e.* low-angle grain boundaries) and *incoherent* boundaries where the disorientation is high (more than 10°). In almost-coherent boundaries, Bain groups from both sides of the boundary are close or may cross each other. Consequently, there is a good agreement between orientations in both grains hinting that, at the present scale, the laths are crossing the original ferrite grain boundary. Fig. 5 ▸ shows such an example. With a disorientation angle of 6.86° between the two ferritic grains, the morphology and crystal orientations are preserved on both sides of the boundary. The pole figures in Figs. 5 ▸(*b*) and 5 ▸(*c*) illustrate the small gap between the two sides given the accuracy of the orientation measurements. More importantly, they demonstrate that, in each grain, austenite orientations lie on the Bain circle given by its ferrite orientation, meaning that a slight rotation occurs in the vicinity of the boundary to comply with this observation.

**Figure 6:**
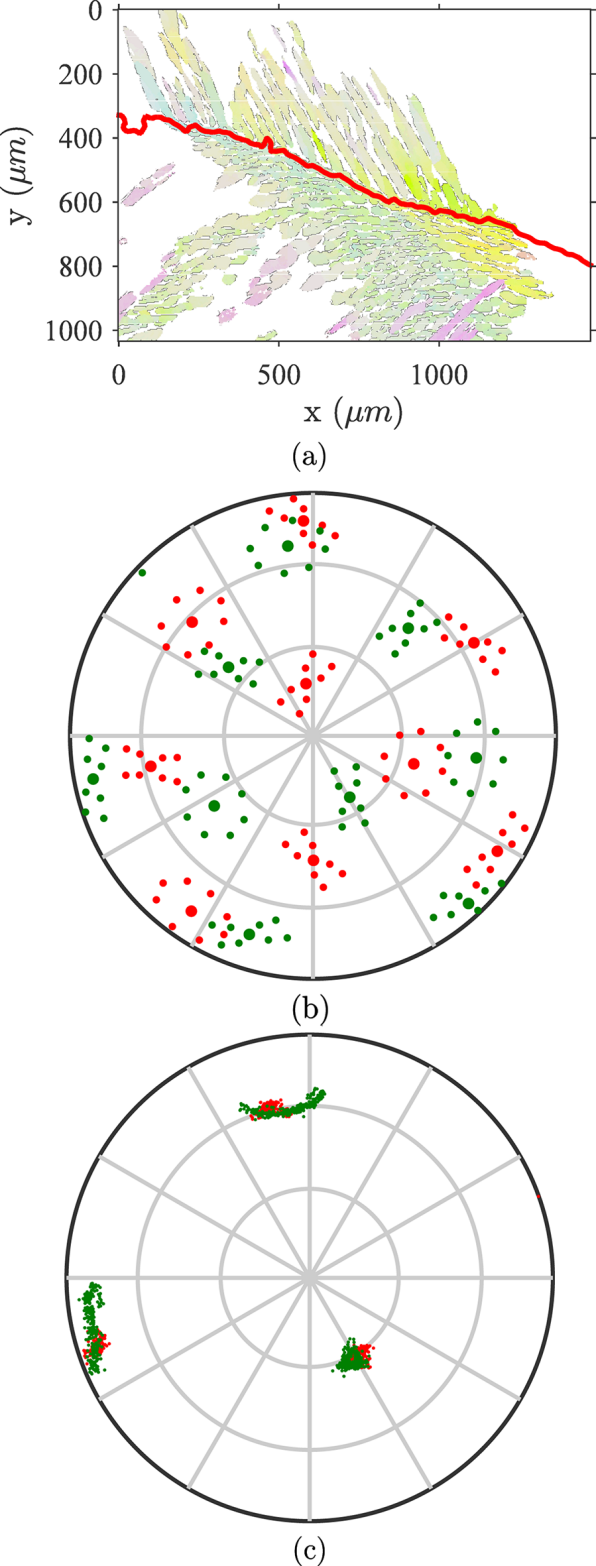
Orientation and morphology for an incoherent boundary. The dis­orientation between the two primary α-grains is 41.1°. (*a*) An IPF-Z map. The primary α-grain boundary is plotted in red. The crystallographic orientation is preserved on both sides of the boundary. In the incoherent grain, a lath shape is also preserved with a different morphological orientation. (*b*) A [100] pole figure of the simulated KS and B variants given the primary α-grain orientations. The red points correspond to the bottom grain and green to the top grain. (*c*) A [100] pole figure showing the preservation of γ-phase orientations in the two parent grains even though it is far from a stable configuration given by standard ORs.

The second type of boundary, referred to as incoherent, corresponds to a large disorientation angle boundary as illustrated in Fig. 6 ▸(*a*). Since the child phase predominantly starts growing from these boundaries, severe accommodation should occur to grow on the side where its orientation does not lie on the corresponding Bain group. Yet the crystallographic orientation is preserved across such boundaries at the cost of a loss of coherence with the parent structure. Fig. 6 ▸(*b*) corresponds to the predicted [100] directions according to the Bain and KS ORs for the two parent grains. Fig. 6 ▸(*c*) reveals the actually measured [100] directions, which are far from the predicted ones for the top grain [red points in Figs. 6 ▸(*b*) and 6 ▸(*c*)] and more coherent with the Bain zone predicted for the bottom grain. However, in the incoherent grain, austenite takes the shape of laths with a different morphological orientation, possibly adapted to the top grain orientation.

## 4. Lath plane determination

### 4.1. Method

The determination of the plane of lath-shaped austenitic crystals (*i.e.* the habit plane) is of great interest to study the local mechanical behavior. Lath clusters have near-uniform or smoothly varying crystallography and morphology. Assuming that these orientations result from a unique mechanism linked to preferential growth directions, there must exist a limited number of configurations to which a relevant homogenized mechanical behavior may be attributed. However, if austenite lath packets are large, their boundaries locally display large fluctuations. This property is likely to be inherited from the long solutionizing heat treatment and prevents the use of the standard habit plane determination methods associated with TEM measurements (Ameyama *et al.*, 1992 ▸; Okamoto & Oka, 1992 ▸; Zhang *et al.*, 1995 ▸; Luo & Liu, 2006 ▸), requiring an accurate and unbiased definition of phase boundaries. Therefore, one may only rely on average measurements to determine the lath extension.

**Figure 7:**
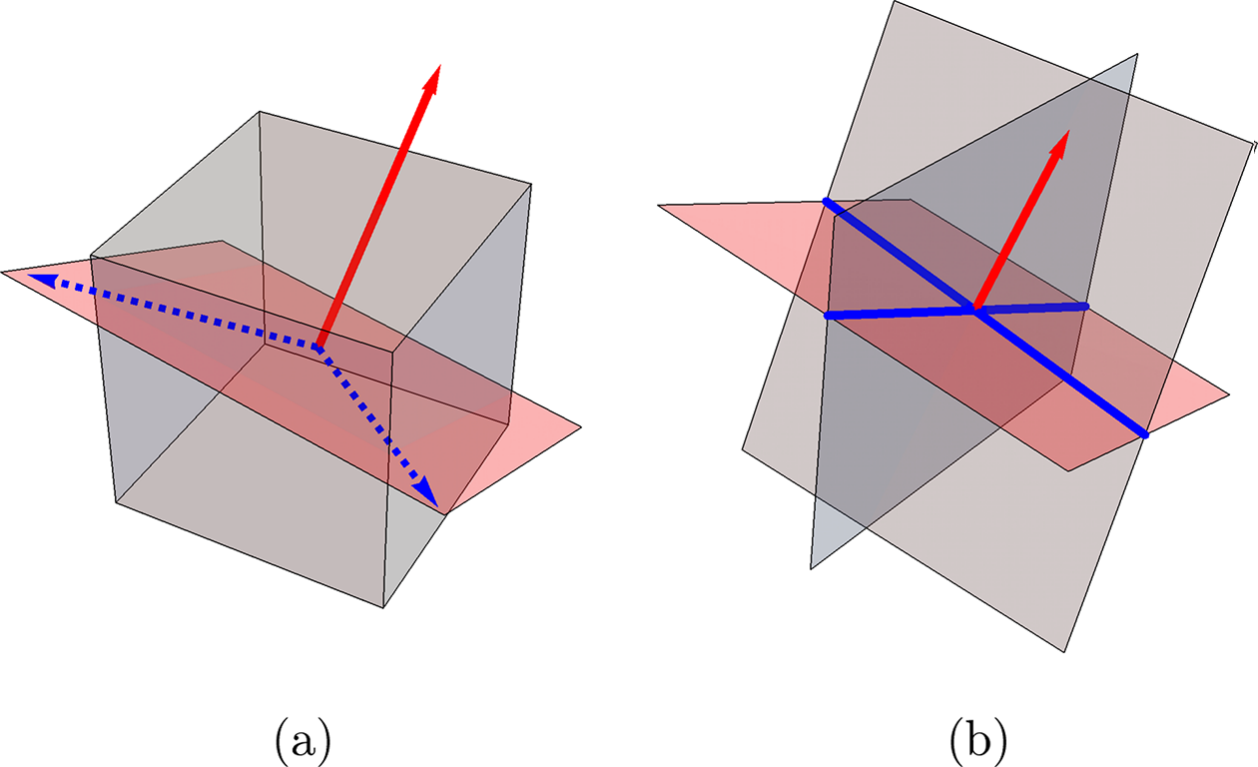
Illustration of the geometric configuration of laths. (*a*) An illustration of a fixed configuration. The lath plane (in red) is fully described by its normal (solid red arrow) or by two non-collinear vectors in the plane (dashed blue arrows). (*b*) The cross section by two observation planes (shaded in gray) gives laths extending along the blue directions (intersection of lath plane and observation plane). Any cross section of the lath plane has a principal direction in two dimensions that is a basis vector of the lath plane in three dimensions. At least two (non parallel) cross sections are required for full determination of the 3D lath normal.

The lath plane orientation in three dimensions is fully described by the orientation of its normal vector. However, as only cross sections with the observation plane are accessible, they cannot provide the 3D lath orientation. Fig. 7 ▸ illustrates the ill-posedness of a direct evaluation of the lath plane normal. However, assuming a unique link between the orientation of the lath morphology and crystallography renders the problem statistically well-posed. As shown in Fig. 7 ▸, two different cross sections offer enough information to infer the lath plane normal. Thus, exploiting the sampling of different lath orientations within one observation plane, and with the assumption of a deterministic relationship between morphology and crystallography, one can identify this relation. This method can be compared to that proposed by Rohrer *et al.* (2004 ▸) but, in the present case, the average orientation of a set of discrete interfaces is computed instead of a distribution. Let us emphasize that defining the average orientation based on raw pixel data avoids the difficult task of having to tune regularization parameters when extracting boundaries from EBSD maps.

The main difficulty of the above identification results from the crystal symmetries and the possible OR variant selection. The proposed methodology is to postulate one possible OR scenario, obtain the most likely lath orientation direction (assigning the closest child variant based on the same variant for each lath packet) and finally check for consistency with the initial postulated OR. Repeating the same approach for all proposed ORs gives a figure of merit for each of them. Hence, an EBSD map can be processed hierarchically to compute (i) the primary ferritic grains, (ii) the child variant clusters inside each primary grain and (iii) the mean morphological orientation with respect to the parent crystallographic 3D frame. However, the mean orientation computation suffers from all the experimental inaccuracies of EBSD maps (definition of phase boundaries according to the imaging technique, irrespective of their natural fluctuations). Thus, a large statistical sampling is needed to retrieve the lath normal for each cluster. The pseudo-code for this evaluation of each OR from the measured orientation map is given in the following algorithm.
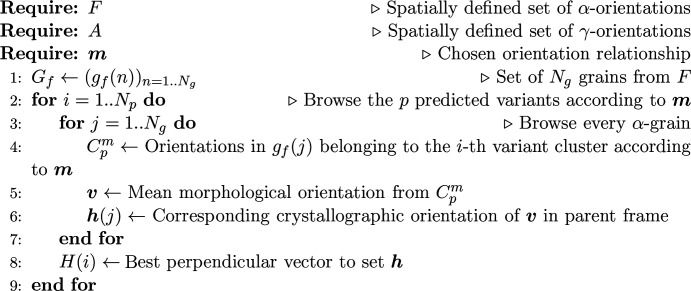


The chosen OR defines the subsets of the orientation map in which the morphological properties are estimated. Each child orientation is attributed to the closest predicted ones considering the local parent orientation and the OR. The mean morphological orientation of each resulting cluster is computed using the structure tensor 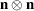, where 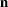 is the mean pixelwise normal vector at the boundary. Its minor eigenvector corresponds to the projection of the mean lath normal in the observation plane, while its major eigenvector relates to the direction of extension of the laths in the packet. Likewise, the ‘best’ perpendicular vector to a set of vectors 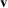 is conventionally defined as the minor eigenvector of 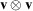.

### 4.2. Application

**Figure 8:**
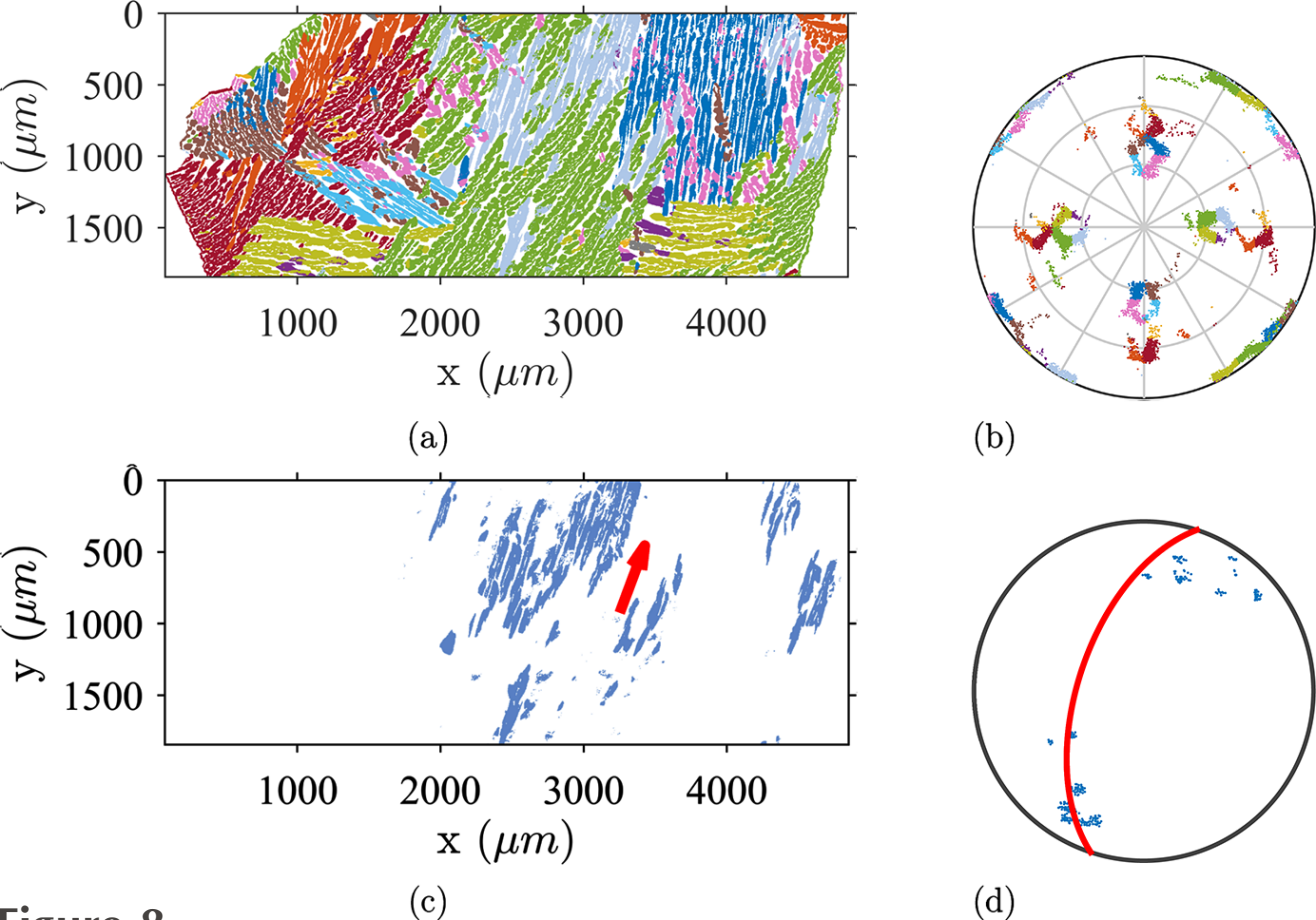
Illustration of the different steps involved in computing the lath normal for the Pitsch OR. (*a*) Austenite orientations colored according to their affiliations to the 12 variants given by the Pitsch OR. (*b*) A [111] pole figure revealing the resulting sampling of the continuous orientation clusters. (*c*) Principal directions computed from pixels belonging to the 12th cluster. (*d*) A spherical projection in the parent frame of principal directions for the 12th variant computed in all processed ferritic grains (blue points) and the resulting ‘mean’ plane trace (red line).

The above construction was tested for all ‘classical’ ORs (*i.e.* B, KS, NW, P and GT). The Pitsch OR appears to provide the most consistent agreement between predicted austenite lath morphology orientation and crystallography. In order to take advantage of a significant statistical distribution, the entire set of acquired maps (140 mm^2^) was used to compute the normals of the 12 child variants predicted by the OR. The different steps are illustrated in Fig. 8 ▸ on the microstructure shown in Figs. 2 ▸ and 4 ▸. Fig. 8 ▸(*a*) shows variant clustering of austenite orientations performed in every ferritic grain according to the Pitsch OR (12 variants). The resulting clusters qualitatively encompass a uniform morphological orientation.

The [111] pole figure [Fig. 8 ▸(*b*)] reveals how the sampling of child orientations given by the associated variant breaks the continuous distribution (introduced in Fig. 3 ▸) into discrete clusters. Once the sampling has been performed, each variant cluster is processed to compute the principal axis of the laths corresponding to the cross section between the lath plane and the observation plane [Fig. 8 ▸(*c*)]. For all processed ferritic grains, this direction is stored as a crystallographic orientation in their frame. These directions should all lie in a plane but some scatter is observed. The laths are not strictly planar and some clusters are not well determined. These differences introduce statistical uncertainties into the principal direction computation. To take into account such errors, the lath plane normal is computed as the ‘best’ (as defined above) perpendicular vector to the set of computed principal directions. As an example, the set of directions and the resulting lath normal for the 12th cluster are shown in Fig. 8 ▸(*d*).

**Figure 9:**
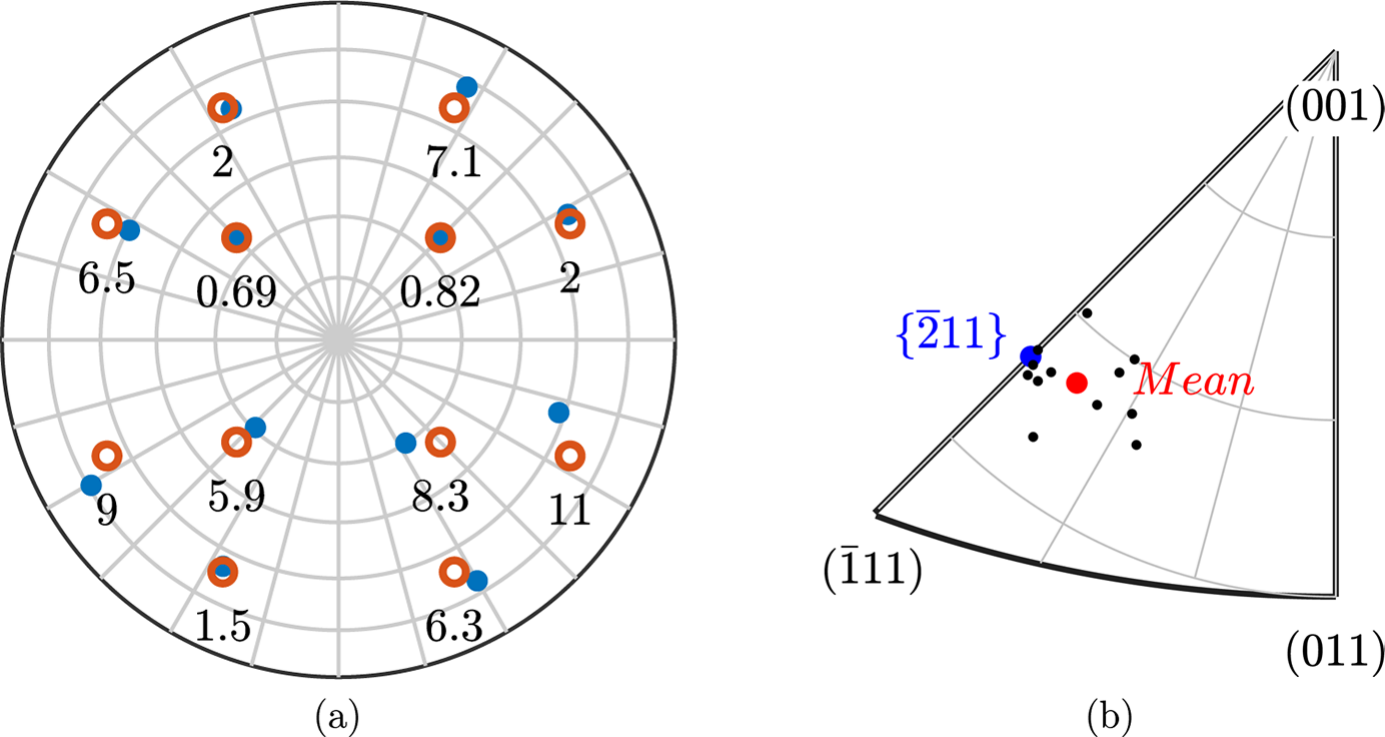
A comparison between Pitsch invariant direction and computed lath normals in the parent frame. (*a*) An element-wise variant comparison. The computed directions are plotted as blue points and 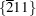 equivalent directions as red circles. The angular error between the two directions is indicated for each variant in degrees. (*b*) A comparison between the equivalent average pole and direction. The 12 computed poles are represented as black points. The average distance between measured and computed poles is 4.1°.

The average lath plane pole in the α frame is given by the direction {−0.996 1.275 2.366}_α_ at about 1° from the rational plane 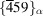 and 4.1° from the invariant direction 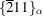 given by the Pitsch OR. The proximity with the latter direction is illustrated in Fig. 9 ▸ by plotting the differences with the computed plane directions and variants of the 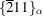 direction given by the Pitsch OR. The standard deviation of the 12 computed poles is 4.45°. This value seems reasonable, considering the limited accuracy of trace-analysis-based techniques on more suitable cases [*e.g.* two-plane analysis on sharply defined twin variants yields an accuracy of around 2° at best; Hoekstra (1980 ▸)].

## 5. Discussion

According to Section 3, the CF8M alloy microstructure in the core of the industrial part is organized hierarchically as follows.

(i) A large equiaxial ferritic grain network resulting from the liquid-to-solid phase transformation. Each grain is encapsulated by a thin austenite layer whose orientation lies on the Bain circle of one of the neighboring grains.

(ii) Lath packets that are either distributed on a Bain circle or attributed to an extension of the γ-orientation that has grown from prior ferritic grain boundaries. Their morphology is inherited from variant selection or induced crystallographic consistency between the two phases. An appropriate orientation–morphology relationship was found by choosing a Pitsch variant clustering.

(iii) Austenite laths themselves described by their crystal orientation and their affiliations to discrete or smoothly varying positions on Bain circles.

The above reading provides a meaningful description of the microstructure from a mechanical point of view. At the observation scale, laths are nearly homogeneous crystals described by their own elasticity and plastic slip directions under mechanical loadings. Lath packets can be modeled by periodic layered domains alternating with two different mechanical responses. The ferritic grains constitute an additional intermediate domain between lath packets and a representative volume that allows one to distinguish packets growing with similar γ-phase orientations on both sides of a prior grain boundary. The mechanical behavior of lath packets is different since the α-phase is oriented differently. Additionally, the lath morphology is most likely to be different on each side to accommodate different growing environments. The dedicated workflow to characterize these morphologies is relevant only for orientations belonging to a specific Bain group since it relies on a variant segmentation.

**Figure 10:**
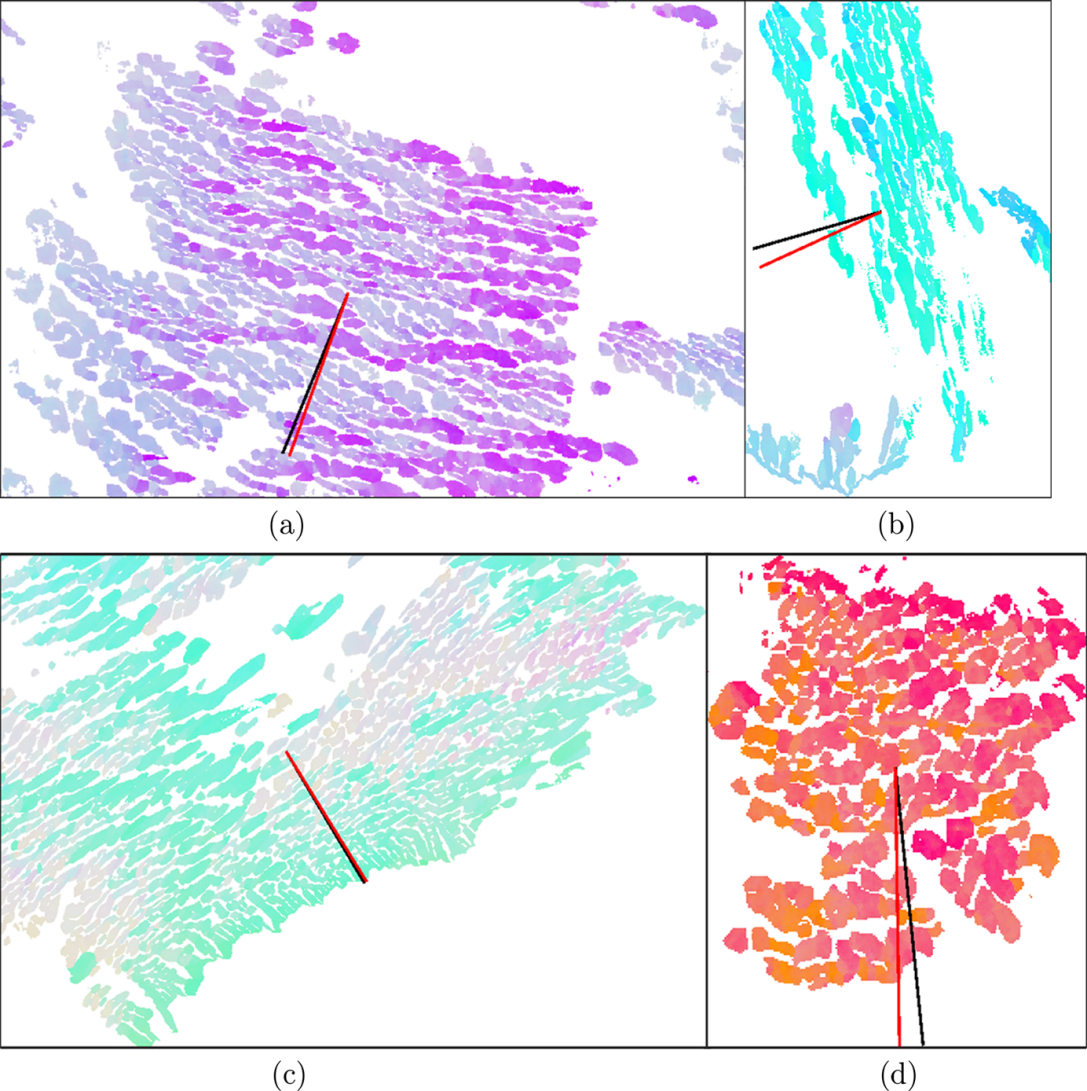
Examples of lath normals in the observation plane as computed on the image (black lines) and invariant 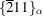 (red lines) directions in representative clusters. The normals are plotted over IPF-Z maps. The figure illustrates the very small difference between the two directions compared with local fluctuations in lath boundaries and the variety of lath cluster morphologies encountered. The angles between the two vectors are (*a*) 2.37°, (*b*) 8.50°, (*c*) 0.98° and (*d*) 4.86°.

While the method may first seem to be of poor accuracy, the results appear to be conclusive in the study of CF8M, especially considering that it only relies on a series of 2D EBSD maps. Interfaces between the two phases at small scales display significant fluctuations, and clusters appear in a wide variety of shapes with different lath morphologies and spatial configurations. At higher scales, a preferential direction of the laths appears more clearly but is to be measured in a similar manner to the one presented herein. The studied slabs are likely to be chemically heterogeneous. Such large ingots exhibit a significant range of cooling rates, and high residual stresses are present. Their redistribution during solutionizing, combined with diffusion mechanisms, adds more distortions from theoretically ideal habit planes. They are most likely deformed by the forming process. Fig. 10 ▸ summarizes the difficulties overcome by the average approach. In most cases, clusters are large enough and characterized with sufficient detail by the EBSD technique to be correctly described with an average definition of the lath normal. This greater part is illustrated in cases (*a*) and (*c*) of Fig. 10 ▸, where the structure tensor gives an admissible normal direction given the visible boundary spread. For these occurrences, the deviation angle between the computed normal and {211}_α_ always falls under 5°, which is consistent with the limited accuracy of EBSD analysis. In more ambiguous cases, including heterogeneous data close to primary grain boundaries [Fig. 10 ▸(*b*)] or when the austenite phase appears more globular on the observation plane [Fig. 10 ▸(*d*)], the prediction is less precise but adequately contributes to the mean direction computation of the whole data set. As a result, the observed gap between theoretically invariant directions and experimentally measured ones is reasonably small (Fig. 10 ▸), and is attributed to experimental deviations of the habit plane and to the out-of-equilibrium configuration due to the long solutionizing treatment.

## 6. Conclusion

Mappings of child orientations on the specimen space and in the parent crystallographic frame allowed us to investigate the mechanisms of the α → γ solid-to-solid phase transformation occurring in the casting process of CF8M steel. The inner structure of the ferritic grains is mainly composed of austenite lath packets growing from their former boundary. Their growth is constrained by the parent grain volume and by simultaneous growth of other packets, resulting in a complex layout. We have shown that the lath packets are reasonably segmented according to their distance to the Bain OR or Pitsch OR variants. Some of the packets belonging to distinct Pitsch variants exhibit the particularity of being spatially connected. This effect manifests itself through smooth crystal and morphological orientation gradients over long characteristic lengths. The morphological orientation was connected to the Pitsch relationship where the child orientation belongs to this OR. A specific method was developed to connect the apparent lath morphology to the local Pitsch variant from the EBSD acquisitions. This then enabled the inference of lath normals in a microstructure with a complex layout. The success of the method proves that austenite lath packets are close to a lath shape. In the present case, it also provided a multiscale segmentation of the microstructure with consistent subsets that are meaningful for understanding the mechanical behavior of the studied dual-phase steel. This process required the parent phase orientation map, and this step was made easier in the CF8M alloy because of the retained parent phase (Mollens *et al.*, 2022 ▸).

The Pitsch OR yielded the best results for determination of the lath properties, even though it is seldomly cited as an OR for f.c.c.–b.c.c. systems. Over the set of 12 computed orientations for lath normals, an uncertainty of about ±2° was reached. This level was deemed precise enough considering the performance of measurements with precisely defined planes on different materials (Hoekstra *et al.*, 1978 ▸; Ameyama *et al.*, 1992 ▸). Multiple factors may lead to a scatter in the locally measured morphology in such large microstructures (*e.g.* varying thermal gradients, chemical segregation or stress accommodation). Hence, an averaging methodology such as the one conducted herein is desirable.

The present study introduced a segmentation of the microstructure relying on morphological and crystallographic aspects of the two phases. The different scales can be segmented and characterized from EBSD data. In view of the lath morphology, the lath cluster scale suggests that significant anisotropy at near-millimetre scale is to be expected. Each scale is embedded in the one above, but the lath packet scale (between ferritic grain scale and lath scale) has broadly scattered characteristic lengths. The packets also inherit some transversely isotropic properties from the lath morphology. This feature is expected to be of great importance in the distribution of strains inside the material. Otherwise, it would mean that crystallographic effects, such as slip transmission between phases, prevail over morphological ones.

The parameters describing lath and lath cluster scales are missing from existing models (Verhaeghe, 1995 ▸; Bugat *et al.*, 1999 ▸; Mcirdl *et al.*, 2001 ▸) and the scale transition rule considered so far does not hold. Instead, the presented results suggest that a sound account of the microstructure at all scales is key to a fair mechanical model. In particular, since aging increases the contrast between phases, such approaches will be needed either to describe plastic flow or to evaluate ‘hot spots’ where stress concentrations will occur, which may trigger microvoids or damage, ultimately leading to fracture.

## Figures and Tables

**Table 1:** Measured chemical composition (wt%) of the ingot from which samples were extracted

C	Si	Mn	S	P	Cr	Ni	Mo	Cu	Co
0.032	1.04	0.80	0.0007	0.025	20.9	10.4	2.68	0.17	0.02

**Table 2:** Orientation relationships commonly referenced between f.c.c. and b.c.c. lattices Both parallelism conditions and axis–angle pairs are given. The latter correspond to the pair having the smallest rotation angle of all the symmetrically equivalent operators.

OR	Plane	Direction	Angle/axis pair
Bain	(001)_γ_ || (001)_α_	[110]_γ_ || [100]_α_	45.0° / [001]
KS	(111)_γ_ || (110)_α_	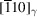 || 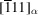	42.9° / [0.97 0.18 0.18]
NW	(111)_γ_ || (110)_α_	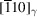 || [001]_α_	46.0° / [0.20 0.98 0.08]
		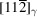 || 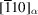	
P	(110)_γ_ || (111)_α_	[001]_γ_ || 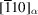	45.0° / [0.08 0.20 0.98]
		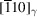 || 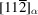	
GT	(111)_γ_ || (110)_α_	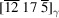 || 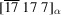	44.2° / [0.97 0.19 0.13]
